# Draft Genome Sequence of the Virulent *Avibacterium paragallinarum* Serotype A Strain JF4211 and Identification of Two Toxins

**DOI:** 10.1128/genomeA.00592-13

**Published:** 2013-08-15

**Authors:** Lisandra Aguilar-Bultet, Sandra P. Calderon-Copete, Joachim Frey, Laurent Falquet

**Affiliations:** CENSA, Centro Nacional de Sanidad Agropecuaria, Mayabeque, Cubaa; Vital-IT, Swiss Institute of Bioinformatics, Lausanne, Switzerlandb; Institute for Veterinary Bacteriology, University of Bern, Bern, Switzerlandc; University of Fribourg and Swiss Institute of Bioinformatics, Fribourg, Switzerlandd

## Abstract

*Avibacterium paragallinarum* is an important pathogen of chicken livestock causing infectious coryza. Here, we report the draft genome sequence of the virulent *A. paragallinarum* serotype A strain JF4211 (2.8 Mbp and G+C content of 41%) and the two toxin operons discovered from the annotation of the genome.

## GENOME ANNOUNCEMENT

Recently, a novel repeat in toxin (RTX) toxin in *Avibacterium paragallinarum* type strain ATCC 29545^T^ and in a virulent *A. paragallinarum* serotype A strain JF4211 was identified (1). To get further information on the virulence attributes of this pathogen, genomic DNA sequencing of strain JF4211 was carried out at the Genome Technology Facility of the University of Lausanne on a Pacific Biosciences (PacBio) machine using standard protocols. The genomic DNA was purified as already published (1), and two libraries of 1 kbp and 10 kbp were prepared according to the manufacturer’s instructions. Each library was run on 2 SMRT cells. The resulting data were quality controlled with FastQC (http://www.bioinformatics.babraham.ac.uk/projects/fastqc/) and assembled on the Vital-IT platform (http://www.vital-it.ch) using the Allora assembly module and the P_ErrorCorrection module of the SMRT pipeline 1.3.3 provided by the manufacturer. The combination of the two libraries of different sizes allowed us to correct for the PacBio huge error rate for large sequences (approximately 15% indels). The draft genome is composed of 34 contigs covering a genome size of 2,869,246 bp. The N_50_ is 168,697 bp, and the minimum and maximum contig sizes are 10,138 and 374,178 bp, respectively. The draft genome was annotated using a pipeline we developed (2) and was deposited at the European Nucleotide Archive (ENA) (3).

The annotation revealed two toxin operons, the AvxA serine-protease RTX toxin (4 genes) (1) and a new cytolethal distending toxin (CDT), which represent potential vaccine targets (4, 5). The RTX toxin is mostly identical to previously described toxins (1, 6) except for the presence of a second potential activator gene in the 5′ end of the operon. Since this gene is separated by a transposase, it might be a reliquary of ancient operon duplication. The cytolethal toxin is similar to previously described toxins (7, 8) in other species, but it has never been described in *A. paragallinarum*. A CDT of *A. paragallinarum* is encoded on 3 genes, *cdtA*, *cdtB*, and *cdtC*, coding for peptides of 214, 275, and 189 amino acids (aa), respectively. The potential ability of *A. paragallinarum* to secrete this cytolethal toxin confirms its pathogenic behavior.

The genes for both toxins were found to be almost identical in the *A. paragallinarum* serotype C strain 72 and serotype A strain 221 (9) available from the NCBI (accession no. AFFP01000000 and AOGF01000000, respectively); thus, both serotypes A and C are very likely to be sensitive to a potential immune response developed against those toxins.

In addition, we also detected the presence of a 6-kbp segment that is very similar to plasmid p250 (10) incorporated into the genome. This is in agreement with a previous work (11) on serotype C, although in our case, an additional integrase gene was found within the plasmid sequence. We assume the latter to play a role in plasmid integration, especially in excision.

### Nucleotide sequence accession numbers.

The sequences of *A. paragallinarum* serotype A strain JF4211 were deposited at EMBL under accession no. CBMK010000001 to CBMK010000034.
